# Editorial: Microbial dysbiosis and immune dysregulation in periodontitis and peri-implantitis

**DOI:** 10.3389/fcimb.2025.1637248

**Published:** 2025-06-20

**Authors:** Ana Carolina Morandini, Pandu R. Gangula, Yuzhou Li, Ke Deng

**Affiliations:** ^1^ Department of Oral Biology, Dental College of Georgia at Augusta University, Augusta, GA, United States; ^2^ Department of Periodontics, Dental College of Georgia at Augusta University, Augusta, GA, United States; ^3^ Department of ODS & Research, School of Dentistry, Meharry Medical College, Nashville, TN, United States; ^4^ Stomatological Hospital of Chongqing Medical University, Chongqing, China; ^5^ Division of Periodontology & Implant Dentistry, Faculty of Dentistry, The University of Hong Kong, Hong Kong, Hong Kong SAR, China

**Keywords:** periodontitis, peri-implantitis, microbial community diversity, immune dysregulation, high-throughput sequencing (deep sequencing)

Microbiome dysbiosis is recognized as one of the drivers of the pathogenesis of periodontitis (Hajishengallis et al., 2012) and peri-implantitis (Kotsakis and Ganesan, 2025). The proliferation of a dysbiotic biofilm together with the dysregulated host immune responses collectively contribute to the break in homeostasis and thus culminate into states of oral diseases (Sedghi et al., 2021).

As part of this Research Topic, the original study by Li et al. employed 16S rDNA sequencing to investigate the microbial communities of infected root canals associated with apical periodontitis in patients with or without diabetes mellitus. Their study sheds light on the diversity and functionality of microbial communities under these two conditions and provides evidence of how type II diabetes can influence the development of periapical disease, particularly from the perspective of microbial communities. In a similar vein, Jia et al. conducted a systematic bioinformatic re-analysis applying high-throughput sequencing of the 16S rRNA gene of microorganisms from the sites of clinical healthy implants, peri-implant mucositis and peri-implantitis. This study provides a comparative analysis between samples from healthy implants and diseased implants, which revealed considerable diversity and richness in the microbiome across the groups.

Regueira-Iglesias et al. in their original study, conducted a 16S multi-batch analysis of approximately 800 saliva samples from both periodontally healthy individuals and those with periodontitis. Their aim was to develop salivary microbiome-based models that can effectively distinguish between periodontal health and diseased states, offering valuable diagnostic tools for accurate diagnosis of periodontitis.

Several review articles in this Research Topic offer diverse perspectives. For instance, Cui et al. review how variations in microbial communities and immune responses contribute to the development of periodontitis and peri-implantitis. They discussed how changes in the oral microbiome - particularly increases in the abundance pathogens such as *Porphyromonas gingivalis*, *Tannerella forsythia* and *Aggregatibacter actinomycetemcomitans*—initiate and progress these diseases. The review not only evaluates current preventive and therapeutic strategies, by emphasizing key microbial and immune factors, but also seeks to inform innovative approaches in the diagnosis, prevention, and treatment of periodontal and peri-implant diseases. Huang et al. provide a comprehensive review article highlighting the impact of microbial dysbiosis and titanium particles in peri-implant microenvironment. They discuss the role of immune dysregulation in peri-implantitis, with a focus on the main inflammatory signaling pathways relevant to the disease.

Contributions from Zhang et al. and Zhang et al. focus on the pathogenic mechanisms and potential applications of bacterial extracellular vesicles as well as nanoparticle-based strategies for enzyme targeting in periodontitis. Both reviews provide updates on recent advances in managing periodontal disease.

Collectively, the original studies and review articles presented in this Research Topic underscore the pivotal role of microbial dysbiosis in the pathogenesis of periodontitis (Lamont et al., 2018) and peri-implantitis, emphasizing the need for targeted therapeutic strategies.
